# Association between a High-Potassium Diet and Hearing Thresholds in the Korean Adult Population

**DOI:** 10.1038/s41598-019-45930-5

**Published:** 2019-07-04

**Authors:** Da Jung Jung, Jae Young Lee, Kyu Hyang Cho, Kyu-Yup Lee, Jun Young Do, Seok Hui Kang

**Affiliations:** 1 Department of Otorhinolaryngology-Head and Neck Surgery, School of Medicine, Kyungpook National University Hospital, Daegu, Republic of Korea; 2 Division of Nephrology, Department of Internal Medicine, Yeungnam University Hospital, Daegu, Republic of Korea

**Keywords:** Nutrition, Epidemiology

## Abstract

The aim of this study was to determine and evaluate the association between potassium intake and hearing thresholds in the Korean adult population. Data from the Korean National Health and Nutrition Examination Survey were analyzed. Participants were divided into tertiles on the basis of their potassium intake as follows: low, middle, and high. Pure-tone audiometry was performed using an automated audiometer. We calculated as the average threshold at the low-frequency pure-tone average (0.5 and 1 kHz), mid-frequency pure-tone average (2 and 3 kHz), and high-frequency pure-tone average (4 and 6 kHz). The average hearing threshold (AHT) was calculated as the pure-tone average of the thresholds at 0.5~3 kHz. Hearing loss (HL) was defined as an AHT of >40 dB in the better ear. There were 1975 participants each in the low, middle, and high tertile groups. The four different average hearing thresholds significantly decreased with an increase in the potassium intake tertile. Multivariate analysis revealed that the four different average hearing thresholds were significantly lower in the high tertile group than in the other two groups. In addition, univariate and multivariate linear regression analyses showed that the potassium intake level was inversely associated with each of the four different average hearing thresholds. Analyses of participants matched based on propensity scores and participants not matched based on propensity scores yielded similar results. The results of this study suggest that high potassium intake levels were associated with a lower prevalence of HL and lower hearing thresholds in the Korean adult population.

## Introduction

Hearing loss (HL) is one of the most common public health problems in the world^1^. With an increase in the life expectancy of the world’s population, many researchers believe that the worldwide prevalence of HL will concurrently increase^2^. WHO revealed that HL affects approximately 5.3% of the world’s population^1^. In particular, a report from New Zealand estimated that the prevalence of HL was approximately 5.08% in 2011, which would increase to 7.02% in 2061^2^. Several studies suggest that various factors, including direct vascular injuries, noise, genetic factors, and environmental exposure, are associated with the development of HL^3–7^. Although some risk factors and interventions have been identified, further research is needed to understand the pathophysiology of HL and determine appropriate prevention strategies.

High-potassium diets are associated with a decrease in or the prevention of various cardiovascular complications^8^. Haijar *et al*. showed that systolic blood pressure is negatively correlated with the amount of potassium intake^9^. A review of 11 cohort studies demonstrated a negative association between potassium intake and strokes^10^. Considering these benefits, the World Health Organization (WHO) recommends a high-potassium diet (at least 3510 mg/day)^11^. Although the benefits of a high-potassium diet have been investigated in many recent studies, its association with various other diseases remains to be elucidated in ongoing research.

Microvascular injury to the cochlea is one of the primary causes of HL^12^. Hwang *et al*. showed that mice with diet-induced obesity had blood vessels with a smaller diameter and thicker walls in the stria vascularis, and this led to hearing impairment^13^. Brown *et al*. investigated the association between a decrease in cochlear vascular reactivity and hearing impairment in old mice^14^. High potassium intake is associated with a decrease in blood pressure through natriuresis and direct vasodilating effects^8,15,16^. Previous epidemiological studies found an inverse relationship between potassium intake levels and the development of hypertension (HTN) and stroke^17,18^. Previous studies showed that various cardiovascular diseases are associated with HL through microvascular injury to the cochlea^19–22^. As high-potassium diets are negatively associated with cardiovascular complications, they may be associated with a decrease in microvascular injury to the cochlea and, consequently, a decrease in the incidence of HL. In addition, previous studies demonstrated that a high-potassium diet is associated with an increase in serum aldosterone levels, which prevents hearing impairment through upregulation of Na^+^-K^+^ ATPase and Na^+^-K^+^-2Cl^−^ cotransporter (NKCC1)^23–27^. From the above perspectives, we conducted the present study to determine and evaluate the association between potassium intake and hearing thresholds in the Korean adult population.

## Materials and Methods

### Study population

Data from the Korean National Health and Nutrition Examination Survey (KNHANES) 2009–2013 were analyzed for this retrospective, cross-sectional study. The KNHANES is a nationwide, multi-stage, stratified survey of a representative sample of the South Korean population conducted by the Korea Centers for Disease Control and Prevention. There were 23 121 participants (n) aged ≥40 years. From these, 17 096 participants were excluded because of missing clinical data and 100 because of an extremely low (<500 kcal/day) or high (>5000 kcal/day) daily energy intake. Finally, 5925 participants were included in this study, which was approved by the Institutional Review Board of Yeungnam University Hospital (IRB number: 2016-10-056). The board waived the need for informed consent because the subjects’ records and information were anonymized and de-identified prior to the analysis. The study was conducted in accordance with the principles that have their origin in the Declaration of Helsinki.

### Study variables

Information collected from the participants during the health examination included the following types of clinical and laboratory data: age, sex, presence of diabetes mellitus (DM), presence of HTN, household income (thousand won/month), smoking habits, alcohol intake, education level, physical activity, estimated glomerular filtration rate (eGFR; mL/min/1.73 m^2^), exposure to explosive noise, exposure to occupational noise, dietary measurements, and hearing thresholds.

DM was defined as a self-reported history of a DM diagnosis, the use of hypoglycemic drugs, a fasting glucose level of ≥126 mg/dL, or an HbA1c of ≥6.5%. HTN was defined as a systolic blood pressure of ≥140 mmHg, a diastolic blood pressure of ≥90 mmHg, a self-reported history of HTN, or the use of anti-HTN drugs. Smokers were classified as current smokers, ex-smokers, or non-smokers. Non-smokers were individuals who had consumed <100 cigarettes in their lifetime, ex-smokers were individuals who had consumed ≥100 cigarettes in their lifetime and had ceased smoking ≥1 year before the survey, and current smokers were individuals who had consumed ≥100 cigarettes in their lifetime and had ceased smoking <1 year before the survey. Alcohol intake was defined by the Korean version of standard drinking, which is based on the WHO classification system^28,29^. We classified alcohol intake into the following categories: abstinence (no alcohol consumption during the 12 months prior to the evaluation), moderate consumption (women, 0.1–19.99 g of pure alcohol/day; men, 0.1–39.99 g of pure alcohol/day), and heavy consumption (women, ≥20 g of pure alcohol/day; men, ≥40 g of pure alcohol/day). Education levels were divided into the following categories: less than high school, high school, and college or more.

Moderate-intensity activities were defined as activities such as job-related activities and light sports such as slow swimming, double tennis, volleyball, badminton, and table tennis. High-intensity activities were defined as activities such as job-related activities and heavy sports such as running, mountain climbing, fast bicycling, fast swimming, soccer, basketball, rope jumping, squash, and single tennis. Regular exercise was defined as engaging in moderate-intensity activity for >30 min/day for 5 days a week or high-intensity activity for >20 min/day for 3 days a week^30^. Physical activity was defined as the performance of regular exercise during leisure time in the 3 months before the evaluation. eGFR was calculated using the Chronic Kidney Disease Epidemiology Collaboration formula^31^.

### Dietary assessments

Dietary assessments were performed by trained staff using a 24-h recall method. A previous study showed a strong correlation between the single 24-h recall method and the multi-step process for each 24-h recall method (Pearson’s correlation coefficients for nutrients was 0.624–0.999)^32^. The total intakes of calories (kcal/day), proteins (g/day), carbohydrates (g/day), fats (g/day), sodium (mg/day), potassium (mg/day), vitamin A (mg/day), carotene (mg/day), retinol (mg/day), thiamine (mg/day), riboflavin (mg/day), niacin (mg/day), and vitamin C (mg/day) were calculated using the nutrient concentrations listed in the Korean Food Composition Table^33^. The total calorie and protein intakes were compared with the recommended amounts for Koreans and were calculated as the proportion of the age- and sex-matched recommended intake for individuals in Korea (%)^34,35^. For example, the calorie intake (%), calculated as the total intake calories/age and sex matched, the recommended total intake calories. Fat and carbohydrate intakes (%) were measured as the distribution of intake calories among the total intake calories (fat intake calories/total intake calories and carbohydrates intake calorie/total intake calories)^35^. The total sodium and potassium intakes were positively associated with the total calorie intake (*r* = 0.538 for sodium and *r* = 0.655 for potassium). Therefore, the sodium and potassium intakes (mg/1000 kcal) were calculated as intakes adjusted for the total calorie intake using the residual method^36^. Subsequently, participants were divided into tertiles on the basis of their potassium intake: low (0–1338 mg/1000 kcal), middle (1339–1761 mg/1000 kcal), and high (1762–10384 mg/1000 kcal). Serum levels of 25-hydroxy-vitamin D_3_ (vitamin D) were measured by a radioimmunoassay (25-hydroxy-vitamin D125 I RIA Kit; DiaSorin, Stillwater, MN, USA) using the 1470 Wizard Gamma Counter (PerkinElmer, Turku, Finland).

### Assessment of hearing thresholds

Histories of exposure to explosive and occupational noise were classified as positive or negative, as previously described^6^. An explosive noise was defined as a sudden loud noise, such as an explosion or gunshot. Exposure to occupational noise was positive if participants had worked in a location with loud machinery for ≥3 months. Exposure to loud noise was positive if participants needed to raise their voice to have a conversation. The hearing thresholds were measured using an automated audiometer at 0.5, 1, 2, 3, 4, and 6 kHz. For both ears of each subject, the threshold values at 0.5 and 1 kHz were averaged to obtain the low-frequency pure-tone average (Low-Freq), the values at 2 and 3 kHz were averaged to obtain the mid-frequency pure-tone average (Mid-Freq), and the values at 4 and 6 kHz were averaged to obtain the high-frequency pure-tone average (High-Freq). In the present study, the average hearing threshold (AHT) was calculated as the pure-tone average of the thresholds at 0.5, 1, 2, and 3 kHz. HL was defined as an AHT of >40 dB in the better ear.

### Statistical analyses

Data were analyzed using SPSS version 21 (SPSS, Chicago, IL, USA). Categorical variables are expressed as numbers and percentages and continuous variables are expressed as means ± standard deviations or standard errors. The distribution of continuous variables was evaluated using the Kolmogorov-Smirnov test. Pearson’s χ^2^ test or Fisher’s exact test was used to analyze categorical variables. For continuous variables, means were compared using *t* -tests or one-way analysis of variance, followed by post-hoc Tukey comparisons. Linear regression analysis was performed to assess the independent predictors of hearing thresholds. Logistic regression analyses were used to estimate the odds ratios and 95% confidence intervals (CIs), which were used to determine the relationship between HL and potassium intake levels.

Multivariate analysis was adjusted for age, sex, DM, HTN, household income, smoking habits, alcohol intake, education level, physical activity, eGFR, calorie intake, protein intake, fat intake, carbohydrate intake, sodium intake, occupational noise exposure, explosive noise exposure, vitamin A intake, carotene intake, retinol intake, thiamine intake, riboflavin intake, niacin intake, vitamin C intake, and serum vitamin D levels. Multivariate analyses were performed using analysis of covariance, multiple linear regression, or multiple logistic regression to determine the independent predictors of hearing thresholds or HL.

Because of substantial differences in the baseline characteristics of the participants in the tertile groups, propensity analysis was performed to minimize bias. To balance the baseline characteristics between the high tertile group and middle and low tertile groups (collectively, non-high group), we estimated propensity scores using logistic regression models and the following variables: age, sex, DM, HTN, household income, smoking habits, alcohol intake, education level, physical activity, eGFR, total energy, protein intake, fat intake, carbohydrate intake, sodium intake, occupational noise exposure, and explosive noise exposure. Participants in the high tertile group were matched with participants in the non-high group using 1:1 nearest neighbor matching without replacement and with a matching tolerance (caliper) of 0.2; the nearest neighborhood matching was based on propensity scores. Before the groups were matched, the standardized mean difference was 0.860, and after matching, the standardized mean difference was 0.094. Before matching, the mean propensity scores for participants in the high tertile group and non-high group were 0.4329 and 0.2836, respectively. After matching, the mean scores were 0.4012 and 0.3848, respectively. A *P*-value of <0.05 was considered statistically significant.

## Results

### Clinical characteristics of the participants

The study population predominantly comprised women (57.1%; Table 1). The mean age was higher for men than for women, and the proportion of participants with DM or HTN was higher among the men than among the women. All nutrient intakes except the sodium intake were higher in men than in women. There were 1975 participants each in the low, middle, and high tertile groups (Table 2). The mean potassium intake in the low, middle, and high tertile groups was 1085 ± 189, 1539 ± 121, and 2262 ± 606 mg/1000 kcal, respectively. The proportion of participants with HTN and that of participants with DM were higher in the low tertile group than in the high tertile group. With an increase in the potassium intake tertile, the rates of HTN, heavy alcohol consumption, occupational noise exposure, and explosive noise exposure and the proportions of men and current smokers decreased, while the carbohydrate intake, sodium intake, and proportion of participants who exercised regularly increased. The mean age and the proportion of participants with DM were the highest in the low tertile group. The household income was the highest in high tertile group, while the education level, eGFR, protein intake, and fat intake were the lowest in the low tertile group. There were significant differences in most baseline characteristics among the tertile groups.

**Table 1:** Clinical characteristics of participants according to sex.

	Total cohort	Men (n = 2542)	Women (n = 3383)	*P*-value
Age (years)	57.6 ± 11.2	58.3 ± 11.4	57.0 ± 11.0	<0.001
Diabetes mellitus	970 (16.4%)	488 (19.2%)	482 (14.2%)	<0.001
Hypertension	2395 (40.4%)	1112 (43.7%)	1283 (37.9%)	<0.001
Household income (thousand won/month)	371 ± 684	377 ± 711	366 ± 664	0.530
Smoking				<0.001
Non-smoker	3598 (60.7%)	439 (17.3%)	3159 (93.4%)	
Ex-smoker	1300 (21.9%)	1200 (47.2%)	100 (3.0%)	
Current smoker	1027 (17.3%)	903 (35.5%)	124 (3.7%)	
Alcohol intake				<0.001
Abstinence	1969 (33.2%)	509 (20.0%)	1460 (43.2%)	
Moderate drinking	3722 (62.8%)	1846 (72.6%)	1876 (55.5%)	
Heavy drinking	234 (3.9%)	187 (7.4%)	47 (1.4%)	
Education level				<0.001
Less than high school	2853 (48.2%)	1002 (39.4%)	1851 (54.7%)	
High school	1848 (31.2%)	830 (32.7%)	1018 (30.1%)	
College or more	1224 (20.7%)	710 (27.9%)	514 (15.2%)	
Physical activity	2799 (47.2%)	1291 (50.8%)	1508 (44.6%)	<0.001
eGFR (mL/min/1.73 m^2^)	89.2 ± 15.0	86.0 ± 15.1	91.6 ± 14.4	<0.001
Calorie intake (%)	99.1 ± 33.8	103.6 ± 34.1	95.7 ± 33.2	<0.001
Protein intake (%)	126.9 ± 64.9	136.4 ± 71.3	119.7 ± 58.7	<0.001
Fat intake (%)	15.3 ± 7.9	15.4 ± 7.7	15.2 ± 8.1	0.231
Carbohydrate intake (%)	68.6 ± 12.7	65.4 ± 13.5	71.0 ± 11.5	<0.001
Sodium intake (mg/1000 kcal)	2306 ± 1288	2360 ± 1294	2266 ± 1283	0.006
Potassium intake (mg/1000 kcal)	1629 ± 611.5	1544 ± 547	1692 ± 648	<0.001
Occupational noise exposure (%)	910 (15.4%)	554 (21.8%)	356 (10.5%)	<0.001
Explosive noise exposure (%)	1582 (26.7%)	1373 (54.0%)	209 (6.2%)	<0.001

The data are expressed as numbers (percentages) for categorical variables and means ± standard deviations for continuous variables. The differences between men and women were tested using *t*-tests for continuous variables and Pearson’s χ^2^ test or the Fisher’s exact test for categorical variables.

Abbreviation: eGFR, estimated glomerular filtration rate.

**Table 2:** Clinical characteristics of participants according to the potassium intake tertile’.

	Low tertile	Middle tertile	High tertile	*P*-value
Age (years)	58.8 ± 11.9	56.8 ± 11.1*	57.1 ± 10.3*	<0.001
Sex (men)	964 (48.8%)	877 (44.4%)	701 (35.5%)	<0.001
Diabetes mellitus	360 (18.2%)	289 (14.6%)	321 (16.3%)	0.009
Hypertension	890 (45.1%)	754 (38.2%)	751 (38.0%)	<0.001
Household income (thousand won/month)	3290 ± 6400	3710 ± 6360	4130 ± 7660*	0.001
Smoking				<0.001
Non-smoker	1074 (54.4%)	1178 (59.6%)	1346 (68.2%)	
Ex-smoker	456 (23.1%)	456 (23.1%)	388 (19.6%)	
Current smoker	445 (22.5%)	341 (17.3%)	241 (12.2%)	
Alcohol intake				<0.001
Abstinence	625 (31.6%)	630 (31.9%)	714 (36.2%)	
Moderate drinking	1207 (61.1%)	1287 (65.2%)	1228 (62.2%)	
Heavy drinking	143 (7.2%)	58 (2.9%)	33 (1.7%)	
Education level				<0.001
Less than high school	1079 (54.6%)	910 (46.1%)	864 (43.7%)	
High school	555 (28.1%)	617 (31.2%)	676 (34.2%)	
College or more	341 (17.3%)	448 (22.7%)	435 (22.0%)	
Physical activity	871 (44.1%)	923 (46.7%)	1005 (50.9%)	<0.001
eGFR (mL/min/1.73 m^2^)	88.0 ± 15.9	89.6 ± 14.8*	90.0 ± 14.0*	<0.001
Calorie intake (%)	101.2 ± 35.9	99.4 ± 32.6	96.8 ± 32.8*^#^	<0.001
Protein intake (%)	116.8 ± 60.8	130.3 ± 61.0*	133.6 ± 71.3*	<0.001
Fat intake (%)	14.7 ± 8.5	16.1 ± 7.8*	15.1 ± 7.4*	<0.001
Carbohydrate intake (%)	66.9 ± 14.8	68.0 ± 11.6*	70.9 ± 11.0*^#^	<0.001
Sodium intake (mg/1000 kcal)	1895 ± 948	2357 ± 1123*	2667 ± 1587*^#^	<0.001
Occupational noise exposure (%)	387 (19.6%)	281 (14.2%)	242 (12.3%)	<0.001
Explosive noise exposure (%)	621 (31.4%)	514 (26.0%)	447 (22.6%)	<0.001
Vitamin A intake (mg/day)	506 ± 531	758 ± 664*	1067 ± 1234*^#^	<0.001
Carotene intake (mg/day)	2483 ± 2531	3890 ± 3507*	5754 ± 6852*^#^	<0.001
Retinol intake (mg/day)	78 ± 195	95 ± 248	108 ± 580*	0.050
Thiamine intake (mg/day)	1.5 ± 0.9	1.6 ± 0.9*	1.8 ± 1.0*^#^	<0.001
Riboflavin intake (mg/day)	1.0 0.6	1.2 ± 0.7*	1.4 ± 0.8*^#^	<0.001
Niacin intake (mg/day)	14.0 ± 8.2	16.3 ± 8.1*	17.0 ± 8.3*^#^	<0.001
Vitamin C intake (mg/day)	59 ± 56	107 ± 88*	165 ± 134*^#^	<0.001
Serum vitamin D levels (ng/mL)	18.8 ± 7.2	18.7 ± 7.0	18.6 ± 6.7	0.846

The data are expressed as numbers (percentages) for categorical variables and means ± standard deviations for continuous variables. The differences between the potassium intake tertiles were tested using one-way analysis of variance for continuous variables, followed by post hoc Tukey comparisons, and Pearson’s χ^2^ test or Fisher’s exact test for categorical variables. *P < 0.05 versus the low tertile group. ^#^*P* < 0.05 versus the middle tertile group.

Abbreviations: eGFR, estimated glomerular filtration rate.

### Association between potassium intake tertile and hearing thresholds

**Figure 1:**
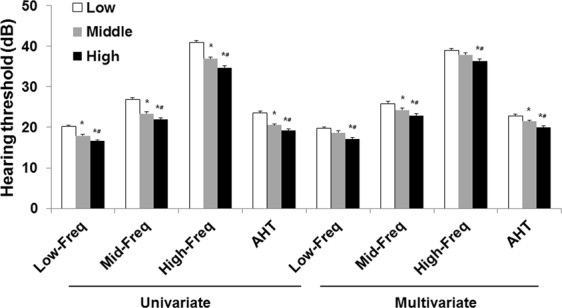
Hearing thresholds according to potassium intake tertiles. Multivariate analysis was adjusted for age, sex, diabetes mellitus, hypertension, household income, smoking habits, alcohol intake, education level, physical activity, eGFR, calorie intake, protein intake, fat intake, carbohydrate intake, sodium intake, occupational noise exposure, explosive noise exposure, vitamin A intake, carotene intake, retinol intake, thiamine intake, riboflavin intake, niacin intake, vitamin C intake, and serum vitamin D levels (*P* < 0.001 for trends in all analyses). The data are expressed as mean and standard error values. *P < 0.05 versus the low tertile group. ^#^*P* < 0.05 versus the middle tertile group. Abbreviations: AHT, average hearing threshold; Low-Freq, low frequency threshold; Mid-Freq, middle frequency threshold; High-Freq, high frequency threshold; eGFR, estimated glomerular filtration rate.

In the low, middle, and high tertile groups, Low-Freq (mean ± standard error) was 20.2 ± 0.4, 17.9 ± 0.3, and 16.6 ± 0.3 dB, respectively (*P* < 0.001 for trend, Fig. 1); Mid-Freq was 26.9 ± 0.4, 23.4 ± 0.4, and 21.9 ± 0.4 dB, respectively (*P* < 0.001 for trend); High-Freq was 40.9 ± 0.5, 36.9 ± 0.5, and 34.7 ± 0.5 dB, respectively (*P* < 0.001 for trend); and AHT was 23.6 ± 0.4, 20.6 ± 0.3, and 14.3 ± 0.3 dB, respectively (*P* < 0.001 for trend). The four different average hearing thresholds significantly decreased with an increase in the potassium intake tertile. Multivariate analysis revealed that the four different average hearing thresholds were significantly lower in the high tertile group than in the low and middle tertile groups. In addition, univariate and multivariate linear regression analyses showed that the potassium intake level was inversely associated with each of the four different average hearing thresholds (Table 3).

**Table 3:** Linear regression analyses of hearing thresholds according to potassium intake levels.

Dependent variables	Univariate		Multivariate	
	Standardized *β* ± SE	*P*-value^*^	Standardized *β* ± SE	*P*-value^*^
Low-Freq	−0.095 ± 0.000	<0.001	−0.057 ± 0.000	<0.001
Mid-Freq	−0.097 ± 0.000	<0.001	−0.034 ± 0.000	0.009
High-Freq	−0.102 ± 0.000	<0.001	−0.025 ± 0.000	0.036
AHT	−0.102 ± 0.000	<0.001	−0.048 ± 0.000	<0.001

^*^The independent variable was potassium intake level, and the multivariate analysis was adjusted for age, sex, diabetes mellitus, hypertension, household income, smoking habits, alcohol intake, education level, physical activity, eGFR, calorie intake, protein intake, fat intake, carbohydrate intake, sodium intake, occupational noise, explosive noise exposure, vitamin A intake, carotene intake, retinol intake, thiamine intake, riboflavin intake, niacin intake, vitamin C intake, and serum vitamin D levels.

Abbreviation: AHT, average hearing threshold; Low-Freq, low frequency threshold; Mid-Freq, middle frequency threshold; High-Freq, high frequency threshold; SE, standard error; eGFR, estimated glomerular filtration rate.

### Association between potassium intake levels and HL

The prevalence of HL in the low, middle, and high tertile groups was 15.3% (n = 302), 11.1% (n = 220), and 7.8% (n = 155), respectively (*P* < 0.001). Univariate logistic regression showed that, compared with those in the high tertile group, participants in the low tertile group exhibited a 2.120-fold (95% CI, 1.727–2.601; *P* < 0.001) higher odds for HL, while those in the middle tertile group exhibited a 1.472-fold (1.186–1.826; *P* < 0.001) higher odds for HL (Table 4). Multivariate analysis revealed that, compared with those in the high tertile group, participants in the low tertile group exhibited a 1.642-fold (1.152–2.340; *P* = 0.006) higher odds for HL, while those in the middle tertile group exhibited a 1.469-fold (1.080–1.998; *P* = 0.014) higher odds for HL. There was no significant difference in the odds ratio between the middle and low tertile groups.

**Table 4:** Logistic regression analyses of hearing loss according to potassium intake levels.

	Univariate		Multivariate^*^	
	OR (95% CI)	*P*-value	OR (95% CI)	*P*-value
Potassium intake				
Middle tertile (vs high tertile)	1.472 (1.186–1.826)	<0.001	1.469 (1.080–1.998)	0.014
Low tertile (vs high tertile)	2.120 (1.727–2.601)	<0.001	1.642 (1.152–2.340)	0.006
Low tertile (vs middle tertile)	1.440 (1.195–1.735)	<0.001	1.194 (0.894–1.593)	0.230
Age (increase 1-year-old)	1.124 (1.114–1.135)	<0.001	1.093 (1.077–1.110)	<0.001
Sex [reference: men (n = 2542)]	0.544 (0.463–0.639)	<0.001	0.566 (0.391–0.821)	0.003
DM [reference: non-DM (n = 970)]	1.953 (1.618–2.356)	<0.001	1.272 (0.980–1.651)	0.070
HTN [reference: non-DM (n = 2395)]	2.039 (1.735–2.397)	<0.001	1.056 (0.847–1.317)	0.626
Household income (increase thousand won/month)	0.997 (0.997–0.998)	<0.001	1.000 (0.999–1.000)	0.169
Smoking [reference: non-smoker (n = 3598)]				
Ex-smoker (n = 1300)	2.162 (1.802–2.593)	<0.001	1.093 (0.766–1.559)	0.624
Current smoker (n = 1027)	1.311 (1.050–1.637)	0.017	1.047 (0.716–1.531)	0.812
Alcohol [reference: abstinence (n = 1969)]				
Moderate alcohol consumption (n = 3722)	0.566 (0.476–0.668)	<0.001	1.033 (0.813–1.314)	0.789
Heavy alcohol consumption (n = 234)	1.087 (0.752–1.572)	0.657	1.901 (1.106–3.266)	0.020
Education [reference: less than high school (n = 2853)]				
High school (n = 1848)	0.315 (0.255–0.387)	<0.001	0.730 (0.546–0.975)	0.033
College or more (n = 1224)	0.157 (0.113–0.217)	<0.001	0.519 (0.836–1.286)	0.003
Physical activity [ref: non-physical activity (n = 2799)]	1.063 (0.906–1.248)	0.453	1.037 (0.836–1.286)	0.741
eGFR (increase 1 ml/min/1.73 m^2^)	0.960 (0.955–0.964)	<0.001	0.996 (0.988–1.005)	0.396
Calorie intake (increase 1%)	0.996 (0.994–0.999)	0.004	0.997 (0.991–1.004)	0.428
Protein intake (increase 1%)	0.994 (0.993–0.996)	<0.001	1.003 (0.998–1.007)	0.236
Fat intake (increase 1%)	0.929 (0.918–0.941)	<0.001	0.991 (0.969–1.014)	0.443
Carbohydrate intake (increase 1%)	1.032 (1.024–1.040)	<0.001	1.011 (0.996–1.027)	0.142
Sodium intake (increase 1 mg/1000 kcal)	1.000 (1.000–1.000)	0.515	1.000 (1.000–1.000)	0.305
Occupational noise exposure [ref: non-exposure (n = 5015)]	1.500 (1.226–1.835)	<0.001	1.519 (1.147–2.012)	0.004
Explosive noise exposure [ref: non-exposure (n = 4343)]	1.563 (1.319–1.852)	<0.001	0.933 (0.720–1.209)	0.602
Vitamin A intake (increase 1 mg/day)	1.000 (0.999–1.000)	<0.001	1.000 (1.000–1.001)	0.200
Carotene intake (increase 1 mg/day)	1.000 (1.000–1.000)	<0.001	1.000 (1.000–1.000)	0.273
Retinol intake (increase 1 mg/day)	0.996 (0.994–0.997)	<0.001	0.999 (0.998–1.000)	0.121
Thiamine intake (increase 1 mg/day)	0.714 (0.644–0.792)	<0.001	0.993 (0.826–1.194)	0.944
Riboflavin intake (increase 1 mg/day)	0.484 (0.416–0.562)	<0.001	1.086 (0.777–1.519)	0.629
Niacin intake (increase 1 mg/day)	0.951 (0.940–0.963)	<0.001	0.988 (0.960–1.017)	0.420
Vitamin C intake (increase 1 mg/day)	0.996 (0.995–0.997)	<0.001	0.999 (0.997–1.001)	0.212
Serum vitamin D levels (increase 1 ng/mL)	1.025 (1.011–1.038)	<0.001	1.002 (0.987–1.016)	0.816

^*^Multivariate analysis for hearing loss was performed using potassium intake tertiles, age, sex, DM, HTN, household income, smoking habits, alcohol intake, education level, physical activity, eGFR, calorie intake, protein intake, fat intake, carbohydrate intake, sodium intake, occupational noise, explosive noise exposure, vitamin A intake, carotene intake, retinol intake, thiamine intake, riboflavin intake, niacin intake, vitamin C intake, and serum vitamin D levels.

Abbreviations: OR, odds ratio; CI, confidence interval; DM, diabetes mellitus; HTN, hypertension; eGFR, estimated glomerular filtration rate.

### Analyses of participants matched based on propensity scores

**Figure 2:**
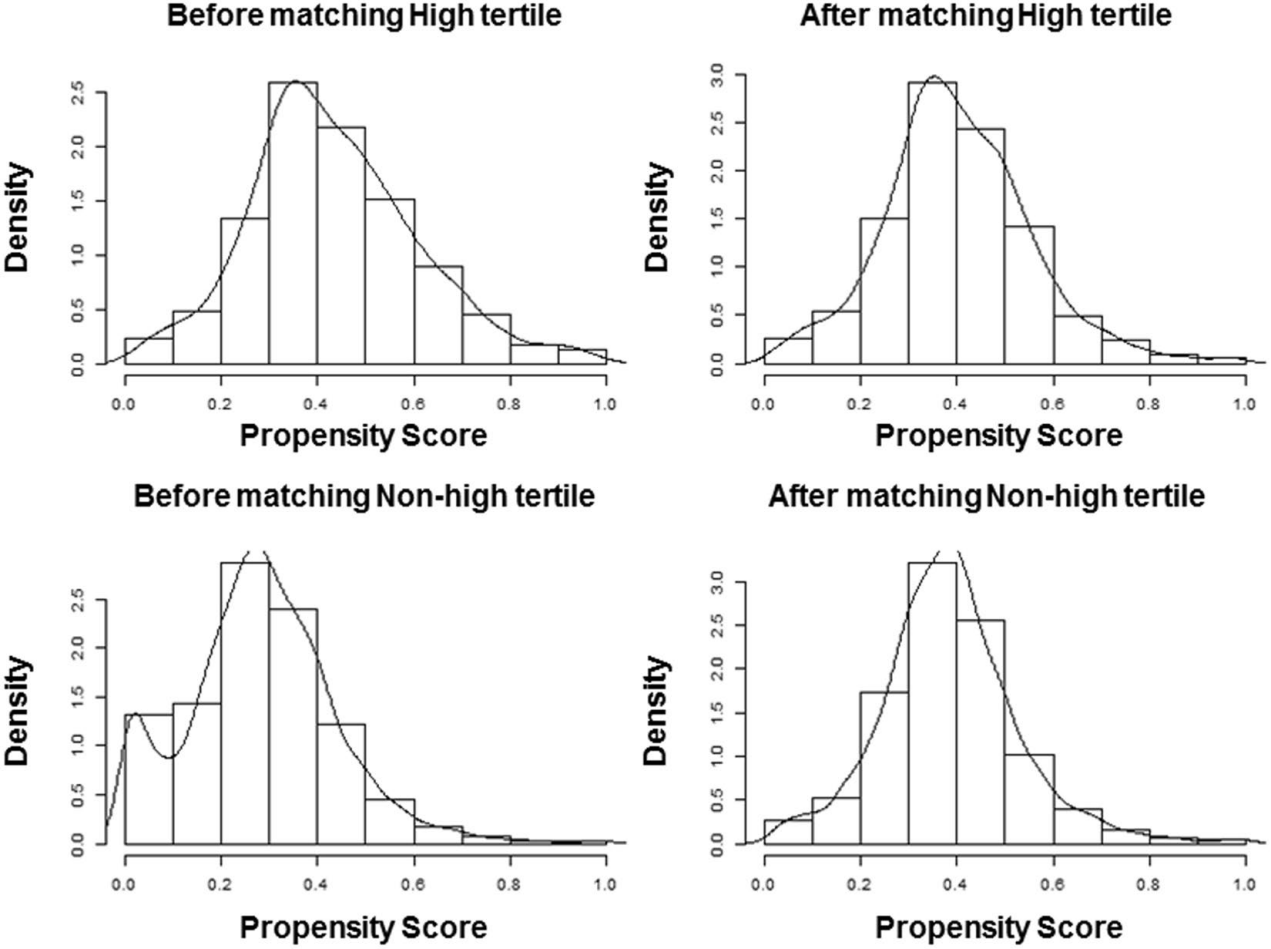
Distribution of propensity scores before and after matching. The distribution of propensity scores before matching differed between the high tertile group and the middle and low tertile groups (collectively, non-high group), although there was no difference after matching.

For propensity score matching, we divided all participants into high tertile and non-high group**s**. In total, 1755 pairs were selected from the 3950 participants in the non-high group. The estimated distribution of propensity scores was similar after matching, and no significant differences in participant characteristics were observed between the high tertile and non-high groups (Fig. 2 and Table 5). Low-Freq in the non-high and high tertile groups was 18.8 ± 15.0 and 16.5 ± 13.1 dB, respectively (*P* < 0.001), Mid-Freq was 24.0 ± 18.5 and 21.9 ± 17.1 dB, respectively (*P* = 0.001), High-Freq was 36.9 ± 22.6 and 34.7 ± 21.2 dB, respectively (*P* = 0.003), and AHT was 21.4 ± 15.9 and 19.2 ± 14.2 dB, respectively (*P* < 0.001). The prevalence of HL in the non-high and the high tertile groups was 12.5% (n = 220) and 7.8% (n = 137), respectively (*P* < 0.001). Univariate logistic regression showed that the participants in the non-high group exhibited a 1.693-fold (95% CI, 1.352–2.119; *P* < 0.001) higher odds of HL compared with those in the high tertile group. Analyses of participants matched based on propensity scores and participants not matched based on propensity scores yielded similar results.

**Table 5:** Clinical characteristics of participants matched by based on propensity scores and grouped by potassium intake.

	Non-high group [middle and low tertile groups] (n = 1755)	High tertile group (n = 1755)	*P*-value
Age (years)	57.2 ± 11.8	57.2 ± 10.3	0.951
Sex (men)	636 (63.8%)	636 (36.2%)	1.000
Diabetes mellitus	272 (15.5%)	281 (16.0%)	0.677
Hypertension	661 (37.7%)	682 (38.9%)	0.466
Household income (thousand won/month)	4010 ± 8620	3930 ± 6150	0.751
Smoking			0.795
Non-smoker	1199 (68.3%)	1181 (67.3%)	
Ex-smoker	333 (19.0%)	347 (19.8%)	
Current smoker	223 (12.7%)	227 (12.9%)	
Alcohol intake			0.230
Abstinence	634 (36.1%)	631 (36.0%)	
Moderate drinking	1079 (61.5%)	1096 (62.5%)	
Heavy drinking	41 (2.4%)	28 (1.6%)	
Education level			0.080
Less than high school	809 (46.1%)	773 (44.0%)	
High school	538 (30.7%)	600 (34.2%)	
College or more	408 (23.2%)	382 (21.8%)	
Physical activity	903 (51.5%)	869 (49.5%)	0.251
eGFR (mL/min/1.73 m^2^)	90.2 ± 14.4	89.8 ± 14.0	0.340
Calorie intake (%)	96.5 ± 32.4	96.3 ± 32.4	0.810
Protein intake (%)	127.4 ± 67.6	128.7 ± 57.4	0.520
Fat intake (%)	15.0 ± 7.7	15.2 ± 7.4	0.598
Carbohydrate intake (%)	70.6 ± 10.9	70.9 ± 10.9	0.390
Sodium intake (mg/1000 kcal)	2493 ± 1264	2452 ± 1274	0.347
Occupational noise exposure (%)	219 (12.5%)	223 (12.7%)	0.839
Explosive noise exposure (%)	400 (22.8%)	404 (23.0%)	0.872

The data are expressed as numbers (percentages) for categorical variables and means ± standard deviations for continuous variables. The *P-*values were tested using *t*-tests for continuous variables and Pearson’s χ^2^ test or Fisher’s exact test for categorical variables.

Abbreviation: eGFR, estimated glomerular filtration rate.

### Subgroup analyses by sex, DM, and HTN

There were significant differences in baseline characteristics between men and women (Table 1). We performed subgroup analyses according to sex, DM, and HTN. The participants in each group (men and women, DM and non-DM, HTN and non-HTN) were divided into three tertiles on the basis of their potassium intake. The number of men in the low, middle, and high tertile groups was 847, 848, and 847, respectively. The four different average hearing thresholds were significantly higher in the low tertile group than in the other tertile groups (Fig. S1). The prevalence of HL in the low, middle, and high tertile groups was 19.0% (n = 161), 14.2% (n = 120), and 11.8% (n = 100), respectively (*P* < 0.001). The number of women in the low, middle, and high tertile groups was 1127, 1128, and 1128, respectively. Mid-Freq and High-Freq were significantly higher in the low tertile group than in the other tertile groups (Fig. S2). Low-Freq and AHT significantly decreased with an increase in the potassium intake tertile. The prevalence of HL in the low, middle, and high tertile groups was 12.2% (n = 138), 7.9% (n = 89), and 6.1% (n = 69), respectively (*P* < 0.001). In addition, univariate linear regression analyses showed that the potassium intake level was inversely associated with each of the four different average hearing thresholds in women and Low-Freq, Mid-Freq, and AHT in men (Table S1).

The number of non-DM participants in the low, middle, and high tertile groups was 1651, 1652, and 1652, respectively. The four different average hearing thresholds significantly decreased with an increase in the potassium intake tertile (Fig. S2). The prevalence of HL in the low, middle, and high tertile groups was 13.6% (n = 224), 10.0% (n = 165), and 6.8% (n = 113), respectively (*P* < 0.001). The number of DM participants in the low, middle, and high tertile groups was 323, 324, and 323, respectively. The four different average hearing thresholds were significantly higher in the low tertile group than in the high tertile group. The prevalence of HL in the low, middle, and high tertile groups was 23.8% (n = 77), 17.3% (n = 56), and 13.0% (n = 42), respectively (*P* = 0.001).

The number of non-HTN participants in the low, middle, and high tertile groups was 1176, 1177, and 1177, respectively. The four different average hearing thresholds were significantly higher in the low tertile group than in the other tertile groups (Fig. S3). The prevalence of HL in the low, middle, and high tertile groups was 12.2% (n = 143), 7.4% (n = 87), and 5.8% (n = 68), respectively (*P* < 0.001). For HTN participants, the numbers of participants in low, middle, and high potassium intake tertiles were 798, 799, and 798, respectively. The four different average hearing thresholds were significantly lower in the high tertile group than in the other tertile groups. The prevalence of HL in the low, middle, and high tertile groups was 19.2% (n = 153), 17.3% (n = 138), and 11.0% (n = 88), respectively (*P* < 0.001).

## Discussion

In the present study, we determined and evaluated the association between potassium intake and hearing thresholds in the Korean adult population. The results indicated an association of potassium intake levels with the four different average hearing thresholds (Low-Freq, Mid-Freq, High-Freq, and AHT) and HL. Univariate and multivariate analyses showed that the four different average hearing thresholds were lower in the high tertile group than in the middle and low tertile groups. Linear regression analyses showed inverse associations between potassium intake levels and the four different average hearing thresholds. Logistic regression analyses showed an association between potassium intake levels and HL.

Various nutrients have been reported to be associated with hearing impairment^6,35,37–39^. Some studies showed an association between factors depicting the general nutrition status, such as body indices or serum albumin levels, and hearing impairment^37^. Using a body composition analyzer, Lee *et al*. showed an association between sarcopenia and hearing impairment^38^. Kang *et al*., who analyzed a cohort similar to ours, showed that the levels of micronutrients, including vitamins C and D, are associated with hearing thresholds^6^. Some reports showed associations between dietary patterns and hearing^35,39^. Prospective or cross-sectional studies examining the association between nutrients and hearing impairment may be on-going.

Potassium is an important electrolyte that plays key roles in cell metabolism and maintenance of the cell membrane potential. The range of normokalemia is narrow (3.5–5.0 mEq/L), and hypokalemia or hyperkalemia can lead to serious complications, such as cardiac arrest or respiratory failure^40^. The kidney is an important organ with a wide excretion rate (5–500 mEq/day), and the development of hyperkalemia or hypokalemia in an individual on a regular diet (35–110 mEq/day) is rare^41^. Several researchers have focused on the association between cardiovascular disease and dietary potassium levels^8–10^. Generally, high-potassium diets reverse the effects of cardiovascular diseases through increases in diuresis and decreases in smooth muscle proliferation, free radicals, and platelet aggregation^41^.

There are few animal or human studies regarding the direct or indirect effects of a high-potassium diet on hearing impairment. The mechanisms underlying the relationship between a high-potassium diet and hearing impairment are poorly understood. However, there are some suggestions regarding this issue. Small changes beyond the normal serum potassium level would be critical. Therefore, favorable results for hearing impairment in individuals on a high-potassium diet may be associated with the secondary effect of a decrease in the extracellular fluid and blood pressure through natriuresis caused by high-potassium diet, rather than changes in the serum potassium level. Previous studies that investigated the association between HTN and cochlear damage provide indirect evidence of the association between a high-potassium diet and hearing impairment. Tachibana *et al*. showed that hypertensive rats exhibited cochlear damage through a decrease in the oxygen supply from the stria vascularis^42^. McDormick *et al*. showed a positive association between cochlear potential and blood pressure^43^.

Another pathogenesis is upregulation of Na^+^-K^+^ ATPase and NKCC1 through aldosterone. Previous studies showed that a high-potassium diet is associated with an increase in aldosterone, which leads to upregulation of Na^+^-K^+^ ATPase in both renal tubules and the stria vascularis^23,24^. Ding *et al*. also showed that aldosterone upregulated NKCC1 in an *in vitro* study^25^. Tardos *et al*. showed that serum aldosterone levels decrease with age in humans and are inversely associated with the severity of presbycusis^26^. In addition, an *in vivo* study showed that long-term aldosterone treatment increases NKCC1 expression, which prevents the progression of age-related HL^27^. Upregulation of Na^+^-K^+^ ATPase and NKCC1 through aldosterone would be helpful in maintaining a proper potassium recycle and endocochlear potential, which may be associated with the preventive effects of a high-potassium diet. Although our study did not analyze aldosterone levels, the abovementioned studies provide indirect evidence regarding the association between a high-potassium diet and hearing impairment.

Steinke *et al*. showed that mutation or post-translational modification of CLC-K/Barttin is important for potassium secretion into the endocochlea, but the incidence of Bartter’s syndrome caused by these abnormalities may be rare in the general population (1.2/million)^44–46^. Abnormalities in CLC-K/Barttin chloride channels combined with clinical significance would be ignored in population-based study.

Dietary patterns are influenced by various characteristics such as age, sex, and socioeconomic status. The clinical effects of diet are small but long-standing, unlike those of other interventions or drugs. These effects may be associated with the differences in baseline characteristics among groups in the present study. Using both multivariate analyses and propensity score matching, we tried to adjust for these differences. The results of multivariate analyses were similar to those of univariate analyses. In addition, we adjusted for the differences in baseline characteristics between the non-high group (middle and low tertile) and the high tertile group using propensity score matching. These analyses showed that the four different average hearing thresholds and HL were both inversely associated with potassium intake levels.

Our study was a retrospective, cross-sectional study, and proper adjustment for confounding factors was a major concern for our study design. There were significant differences in baseline characteristics among the potassium tertile groups. Although multivariate analysis is useful for adjusting different characteristics, additional data regarding propensity analysis would be helpful to overcome these confounding factors or differences in baseline characteristics. In the present study, propensity scores were calculated between the high tertile and non-high groups, and participants matched based on propensity scores were analyzed.

In the present study, participants were divided into tertile groups according to potassium intake levels. A dichromatic approach, such as the inclusion of normal and abnormal groups, will be prone to false-positive results. Analyses using more categories would be helpful to identify changes in hearing according to potassium intake levels; however, several categories will be associated with complex results that may be difficult to understand. We used tertile levels in the present study, which were easy to understand and minimized false-positive results.

This study had a few limitations. Firstly, it was a cross-sectional design with a failure to establish causality. Second, the study evaluated potassium intake levels using the food intake survey and the 24-h recall method, and possible errors in potassium measurements may have biased the results. Third, between groups, statistically significant differences in hearing thresholds were small. Fourth, there is possibility of a selection bias because of the exclusion of 17 096 participants with missing data. The excluded participants represented approximately 74.0% of the population. In particular, data regarding hearing thresholds was missing for 13 338 participants. Our study did not perform additional analyses regarding missing data. Fifth, in our study, multivariate analyses were adjusted for many, but not all confounding factors. Possible confounding factors, such as history of ototoxic drug use, recent trauma, genetic susceptibility to hearing impairment, or omega-3 fatty acid intake, were not considered in the present study. Further prospective studies that include follow-up data, more confounding factors, and speech discrimination are necessary to overcome selection bias and evaluate a possible correlation between potassium intake levels and hearing impairment. Including speech discrimination can not only help to confirm hearing impairment, but also to understand the effect it has in daily life.

In conclusion, high potassium intake levels were associated with a lower prevalence of HL and lower hearing thresholds in the Korean adult population. Further research should determine whether interventions that improve the potassium intake may help in preventing hearing impairment in the Korean adult population.

## Supplementary information

Supplementary Information
