# Supplementary material for: Association between a High-Potassium Diet and Hearing Thresholds in the Korean Adult Population
Source: Sci Rep. 2019 Jul 4;9:9694. doi: 10.1038/s41598-019-45930-5 (PMC6609769; doi:10.1038/s41598-019-45930-5)
Supplement: Supplementary file 1 — Supplementary Information [file 41598_2019_45930_MOESM1_ESM.docx]

**Association between a High-Potassium Diet and Hearing Thresholds in the Korean Adult Population**

Da Jung Jung, MD, PhD^1^, Jae Young Lee, MD^1^, Kyu Hyang Cho, MD, PhD^2^,

Kyu-Yup Lee, MD, PhD^1^, Jun Young Do, MD, PhD^2^, and Seok Hui Kang, MD, PhD^2^

^1^**Department of Otorhinolaryngology-Head and Neck Surgery, School of Medicine, Kyungpook National University Hospital, Daegu, Republic of Korea**

^2^Division of Nephrology, Department of Internal Medicine, Yeungnam University Hospital, Daegu, Republic of Korea

**Corresponding author**: Seok Hui Kang, MD, Department of Internal Medicine, Yeungnam University Hospital, 317-1 Daemyung-Dong, Nam-Ku, Daegu 705-717, Korea

Fax: +82-53-623-8180, Phone: +82-53-6620-3347, E-mail: kangkang@ynu.ac.kr

**Running title**: Association between Potassium Intake and Hearing

**Funding and Support statement**: This study was supported by the Medical Research Center Program through the National Research Foundation of Korea (NRF), funded by the Ministry of Science, ICT and Future Planning [grant number 2015R1A5A2009124] and the NRF Grant funded by the Korea government (MSIT) [grant number 2018R1C1B6007775]. The funders had no role in study design, data collection and analysis, decision to publish, or preparation of the manuscript.

**Competing Interests**: The authors have declared that no competing interests exist.

**Author contributions**: SHK and JYD conceived the research idea; KHC and JYL performed the experiments and the statistical analyses; DJJ and SHK wrote the paper; JYD and SHK had primary responsibility for final content. All authors approved the final version of the manuscript.

**Supplementary Information**

Figure S1: Hearing thresholds according to potassium intake tertiles in men and women.

Figure S2: Hearing thresholds according to potassium intake tertiles in participants with and without DM.

Table S1: Linear regression analyses of hearing thresholds according to potassium intake levels in men and women.

Figure S3: Hearing thresholds according to potassium intake tertiles in participants with and without HTN.


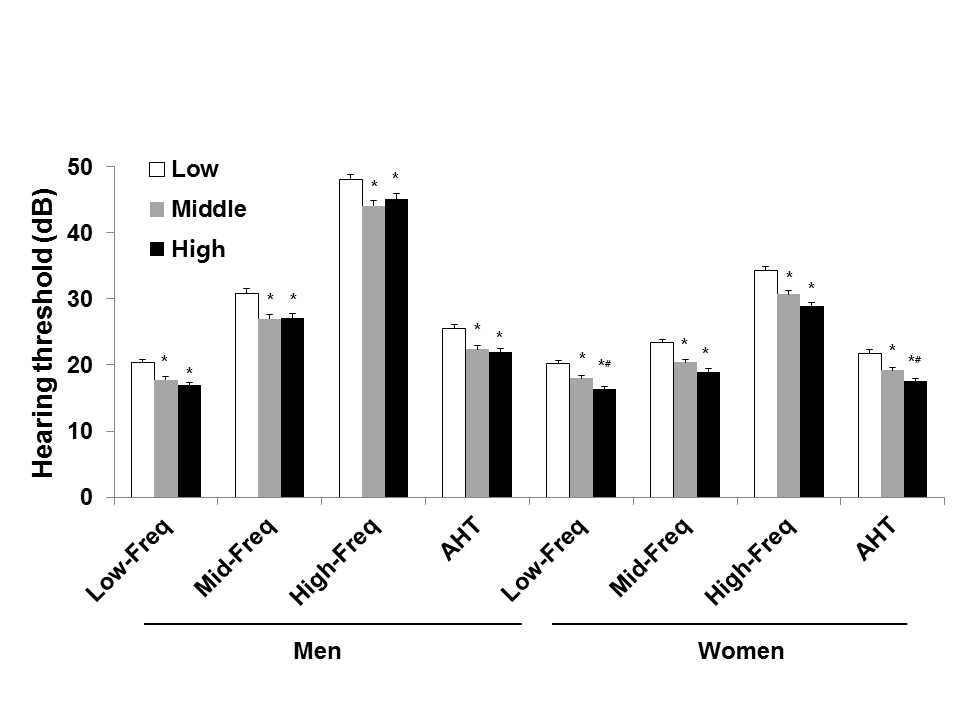
**Figure S1. Hearing thresholds according to potassium intake tertiles in men and women.** For men, the mean hearing thresholds in the low, middle, and high tertile groups were as follows: Low-Freq, 20.4 ± 0.5 dB, 17.8 ± 0.5 dB, and 16.9 ± 0.5 dB, respectively; Mid-Freq, 30.9 ± 0.7 dB, 27.0 ± 0.7 dB, and 27.1 ± 0.7 dB, respectively; High-Freq, 48.1 ± 0.8 dB, 44.1 ± 0.8 dB, and 45.1 ± 0.8 dB, respectively; and AHT, 25.6 ± 0.6 dB, 22.4 ± 0.5 dB, and 22.0 ± 0.5 dB, respectively. For women, the mean hearing thresholds in the low, middle, and high tertile groups were as follows: Low-Freq, 20.2 ± 0.5 dB, 18.0 ± 0.4 dB, and 16.3 ± 0.4 dB, respectively; Mid-Freq, 23.4 ± 0.5 dB, 20.4 ± 0.5 dB, and 19.0 ± 0.4 dB, respectively; High-Freq, 34.3 ± 0.6 dB, 30.7 ± 0.6 dB, and 28.9 ± 0.5 dB, respectively; and AHT, 21.8 ± 0.5 dB, 19.2 ± 0.4 dB, and 17.6 ± 0.4 dB, respectively. The data are expressed as means and standard error values. **P* < 0.05 versus the low tertile group. ^#^*P* < 0.05 versus the middle tertile group. Abbreviations: AHT, average hearing threshold; Low-Freq, low frequency threshold; Mid-Freq, middle frequency threshold; High-Freq, high frequency threshold.


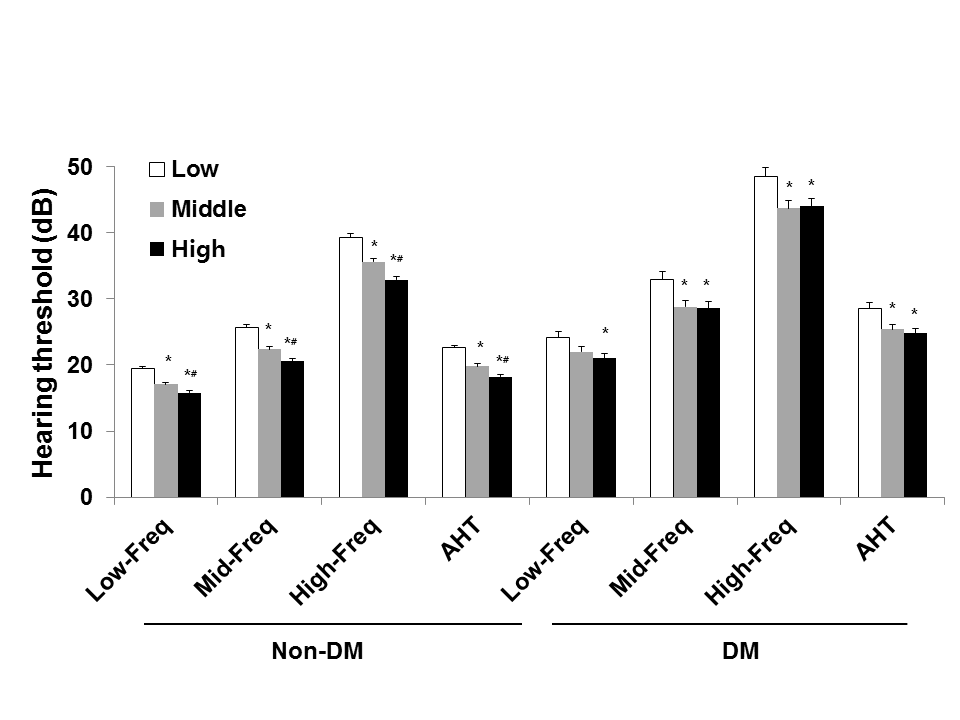


**Figure S2.** **Hearing thresholds according to potassium intake tertiles in participants with and without DM.** For non-DM participants, the mean hearing thresholds in the low, middle, and high tertile groups were as follows: Low-Freq, 19.4 ± 0.4 dB, 17.1 ± 0.3 dB, and 15.8 ± 0.3 dB, respectively; Mid-Freq, 25.7 ± 0.5 dB, 22.4 ± 0.4 dB, and 20.6 ± 0.4 dB, respectively; High-Freq, 39.3 ± 0.6 dB, 35.6 ± 0.5 dB, and 32.9 ± 0.5 dB, respectively; and AHT, 22.6 ± 0.4 dB, 19.8 ± 0.4 dB, and 18.2 ± 0.3 dB, respectively. For DM participants, the mean hearing thresholds in the low, middle, and high tertile groups were as follows: Low-Freq, 24.1 ± 0.9 dB, 22.0 ± 0.8 dB, and 21.0 ± 0.8 dB, respectively; Mid-Freq, 32.9 ± 1.2 dB, 28.8 ± 1.0 dB, and 28.6 ± 1.0 dB, respectively; High-Freq, 48.6 ± 1.3 dB, 43.7 ± 1.2 dB, and 44.0 ± 1.2 dB, respectively; and AHT, 28.5 ± 1.0 dB, 25.4 ± 0.8 dB, and 24.8 ± 0.8 dB, respectively. The data are expressed as means and standard error values. **P* < 0.05 versus the low tertile group. ^#^*P* < 0.05 versus the middle tertile group. Abbreviations: DM, diabetes mellitus; AHT, average hearing threshold; Low-Freq, low frequency threshold; Mid-Freq, middle frequency threshold; High-Freq, high frequency threshold.

**Table S1. Linear regression analyses of hearing thresholds according to potassium intake levels in men and women.**

| **Dependent variables** | **Men** | |  | **Women** | |  |
| --- | --- | --- | --- | --- | --- | --- |
|  | **Standardized *β* ± SE** | ***P*-value** |  | **Standardized *β* ± SE** | ***P*-value** | |
| Low-Freq | –0.071 ± 0.001 | <0.001 |  | –0.110 ± 0.000 | <0.001 | |
| Mid-Freq | –0.049 ± 0.001 | 0.013 |  | –0.098 ± 0.000 | <0.001 | |
| High-Freq | –0.025 ± 0.001 | 0.213 |  | –0.101 ± 0.000 | <0.001 | |
| AHT | –0.062 ± 0.001 | 0.002 |  | –0.108 ± 0.000 | <0.001 | |

Abbreviation: AHT, average hearing threshold; Low-Freq, low frequency threshold; Mid-Freq, middle frequency threshold; High-Freq, high frequency threshold; SE, standard error.

**
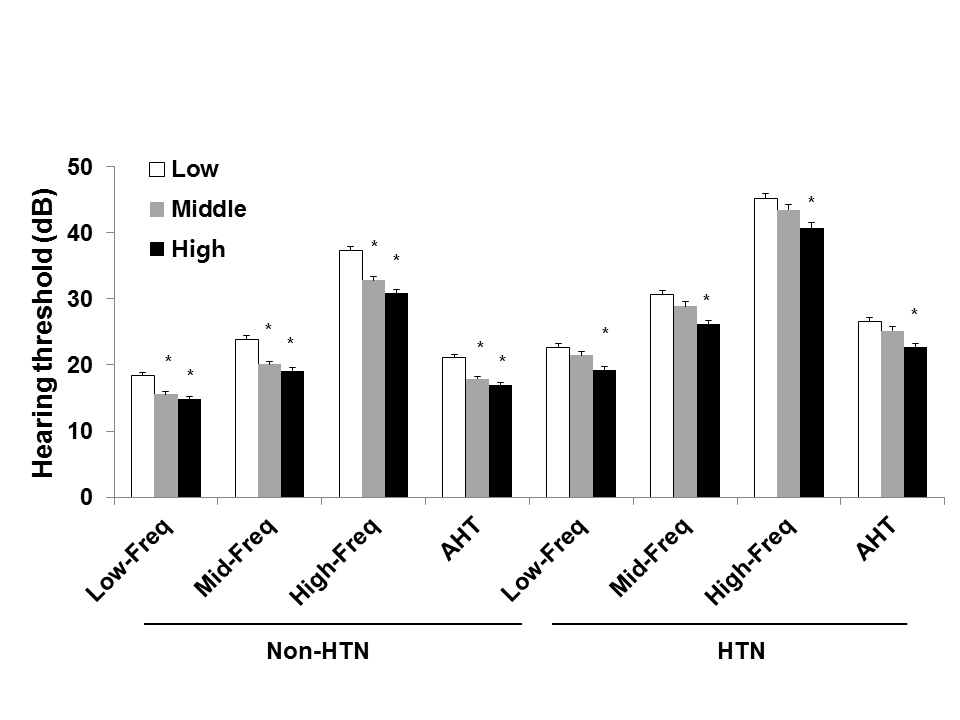
**

**Figure S3. Hearing thresholds according to potassium intake tertiles in participants with and without HTN.** For non-HTN participants, the mean hearing thresholds in the low, middle, and high tertile groups were as follows: Low-Freq, 18.4 ± 0.4 dB, 15.6 ± 0.4 dB, and 14.9 ± 0.4 dB, respectively; Mid-Freq, 23.9 ± 0.6 dB, 20.1 ± 0.5 dB, and 19.1 ± 0.5 dB, respectively; High-Freq, 37.3 ± 0.7 dB, 32.8 ± 0.6 dB, and 30.9 ± 0.6 dB, respectively; and AHT, 22.1 ± 0.5 dB, 17.9 ± 0.4 dB, and 17.0 ± 0.4 dB, respectively. For HTN participants, the mean hearing thresholds in the low, middle, and high tertile groups were as follows: Low-Freq, 22.6 ± 0.6 dB, 21.5 ± 0.5 dB, and 19.2 ± 0.5 dB, respectively; Mid-Freq, 30.6 ± 0.7 dB, 28.9 ± 0.7 dB, and 26.2 ± 0.6 dB, respectively; High-Freq, 45.2 ± 0.8 dB, 43.5 ± 0.8 dB, and 40.7 ± 0.8 dB, respectively; and AHT, 26.6 ± 0.6 dB, 25.2 ± 0.6 dB, and 22.7 ± 0.5 dB, respectively. The data are expressed as means and standard error values. **P* < 0.05 versus the low tertile group. Abbreviations: HTN, hypertension; AHT, average hearing threshold; Low-Freq, low frequency threshold; Mid-Freq, middle frequency threshold; High-Freq, high frequency threshold.
